# Identification of FGF19 as a prognostic marker and potential driver gene of lung squamous cell carcinomas in Chinese smoking patients

**DOI:** 10.18632/oncotarget.7817

**Published:** 2016-03-01

**Authors:** Qiang Tan, Fan Li, Guan Wang, Weiliang Xia, Ziming Li, Xiaomin Niu, Wenxiang Ji, Hong Yuan, Qiang Xu, Qingquan Luo, Jie Zhang, Shun Lu

**Affiliations:** ^1^ Shanghai Lung Cancer Center, Lung Cancer Research Lab, Shanghai Chest Hospital Affiliated to Shanghai Jiao Tong University, Shanghai 200030, China; ^2^ State Key Laboratory of Oncogenes and Related Genes, School of Biomedical Engineering, Shanghai Jiao Tong University, Shanghai 200030, China; ^3^ Genomics Center, WuXi AppTec Co., Ltd., Shanghai 200131, China; ^4^ Jiahui International Hospital, Shanghai 200120, China

**Keywords:** FGF19, lung squamous cell carcinomas

## Abstract

Comprehensive genomic characterizations of lung squamous cell carcinoma (LSCC) have been performed, but the differences between smokers (S-LSCC) and never smokers (NS-LSCC) are not clear, as NS-LSCC could be considered as a different disease from S-LSCC. In this study we delineated genomic alterations in a cohort of 21 NS-LSCC and 16 S-LSCC patients, and identified common gene mutations and amplifications as previously reported. Inclusion of more NS-LSCC patients enabled us to identify unreported S-LSCC- or NS-LSCC-specific alterations. Importantly, an amplification region containing FGF19, FGF3, FGF4 and CCND1 was found five-times more frequent in S-LSCC than in NS-LSCC. Amplification of FGF19 was validated in independent LSCC samples. Furthermore, FGF19 stimulated LSCC cell growth *in vitro*. These data implicate FGF19 as a potential driver gene in LSCC with clinic characteristics as smoking.

## INTRODUCTION

Lung cancer is the most frequent cause of cancer incidence and mortality worldwide. After adenocarcinoma, lung squamous cell carcinoma (LSCC) is the second most common type of lung cancer. Currently no targeted drug is approved for the treatment of LSCC largely due to the lack of knowledge of its molecular pathogenesis. Cigarette smoking is regarded as the most important risk factor for lung cancer, especially for LSCC (accounting for at least 90% patients) [1, 2]. Herein we used the term ‘never smoker’ to describe an individual with lifetime exposure of less than 100 cigarettes, and ‘smoker’ to refer to whoever was either a former or current smoker. An increasing number of ‘never smokers’ were being diagnosed with lung cancer, in which adenocarcinoma was the most common form [3]. Major gender, clinicopathological and molecular differences in lung adenocarcinomas arising in never smokers and in smokers were reported [4]. LSCC in never smokers (NS-LSCC) should be considered as a different disease than those in smokers (S-LSCC) [3, 5]. Recently both the TCGA [6] and Korean [7] studies had characterized LSCC by integrative and comparative genomics approaches. However, never smokers represented less than 5% of the total patients in their cohorts, which could limit effective comparison between S-LSCC and NS-LSCC. Current knowledge of molecular profiles of NS-LSCC is lacking.

Herein, we have conducted a comprehensive study of Chinese LSCC patients, particularly with increased number of never smokers. Our aims are to characterize the genomic landscape differences between S-LSCC and NS-LSCC, and also to identify potential opportunities for therapy.

## RESULTS AND DISCUSSION

As NS-LSCC patients are relatively rare, and the previous two studies included only a few never smokers in their cohorts [6, 7], we intended to collect at least 20 NS-LSCC patients in our study. Twenty-one NS-LSCC patients were enrolled in Shanghai Chest Hospital from April 2009 to December 2012, while 16 S-LSCC patients were included in our study (Table 1; Supplementary Table S1). Tumor samples were reviewed by independent pathologists, from which cancerous and adjacent non-cancerous tissues were subjected to whole-exome sequencing and RNA sequencing followed by key target validations. Customized sequence exome capture methods and bioinformatics analysis were used to identify known and novel genomic aberrations in LSCC, especially those unique to S-LSCC or NS-LSCC. Whole exome sequencing of our cohort identified a total 7,781 somatic mutations, and 79 somatic insertions and deletions events among 21 NS-LSCC and 16 S-LSCC tumor/normal pairs in coding regions. The mean sequencing depth in the targeted bases was 97 folds, with 92.55% of target bases above 30 fold coverage. Individuals display a mean point mutation rate of 3.88 mutations per megabase (Mb) and a median of 3.49 per Mb. This rate is higher than those observed in TCGA projects including acute myelogenous leukemia (0.56 per Mb), breast carcinoma (0.56 per Mb), ovarian cancer (2.1 per Mb) and glioblastoma multiforme (2.3 per Mb); but lower than rates observed in the TCGA LSCC project (8.1 per Mb) [6] and the Korean LSCC study (8.7 per Mb) [7]. The main reason for lower mutation rate in our study is that 21 out of 37 patients are never smokers in our cohort, and mutations in NS-LSCC are rarer. At CpG sites transitions and transversions were the most commonly observed mutation types with mean rates of 9.1 per Mb of CpG context in S-LSCC *versus* 5.7 per Mb in NS-LSCC.

**Table 1:** Clinical data summary

variable	*n* = 37	
	No.	%
**Age at surgery, years**		
Median	61	
Range	40–76	
**Sex**		
Male	32	86.5
Female	5	13.5
**Smoking status**		
Never-smoker	21	56.8
smoker	16	43.2
Former smoker, pack-years	5	13.5
≤ 20	0	0
> 20	5	13.5
**Current smoker, pack-years**	11	29.7
≤ 20	0	0
> 20	11	29.7
Median follow-up, months	35.3	
**Tumor stage**		
I	13	35.1
II	14	37.8
III	10	27.0
IV	0	0
**T stage**		
T1	4	10.8
T2	26	70.3
T3	6	16.2
T4	1	2.7
**N stage**		
N0	19	51.4
N1	10	27.0
N2	8	21.6
N3	0	0

### Somatically mutated genes in both S-LSCC and NS-LSCC exhibit recurrent mutations previously reported in LSCC

**Figure 1:**
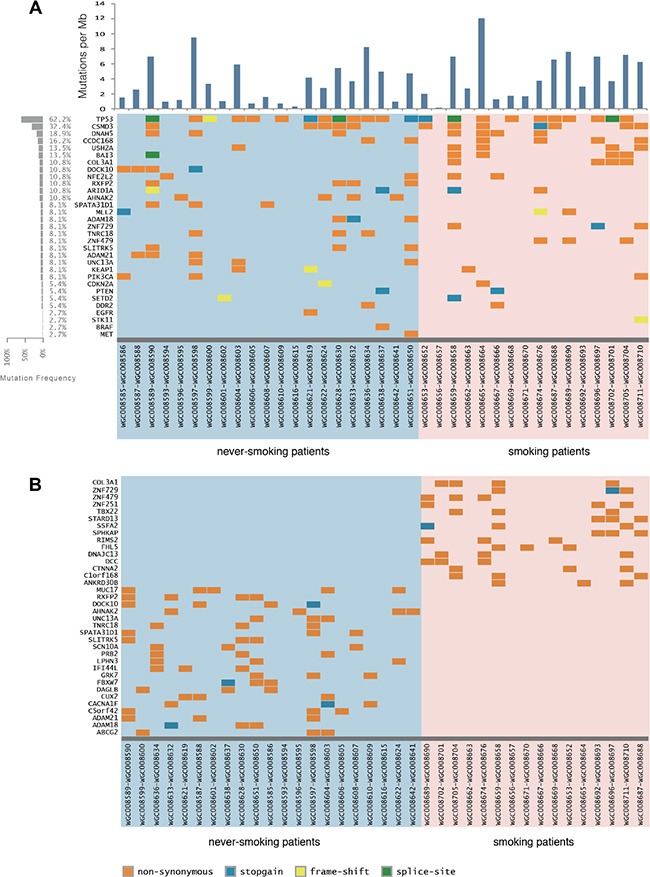
Mutation landscape in lung squamous cell carcinoma (LSCC) of smoking and never-smoking patients (**A**) A heat map of significant genetic events in 37 LSCC patients is provided for both genes previously implicated in lung squamous cell carcinoma and novels genes found to be recurrently altered in the present study. Events, including non-synonymous, stop-gain point mutations and truncation mutations are color coded according to the legend provided. Each column denotes an individual normal/tumor paired patients, and each row represents a gene. The left panel shows the mutation frequency in each gene. (**B**) A similar heat map of mutations found only in smokers or never-smokers.

We first assessed the 37 LSCC samples as a whole and compared the general features in gene mutations with two published reports (Figure 1A; Supplementary Table S2). Comparative analysis between Korean and North American LSCC samples demonstrated a similar spectrum of alterations in these two populations [6, 7] in contrast to the differences seen in lung adenocarcinoma. We identified 17 highly recurrent mutated genes in both S-LSCC and NS-LSCC, including seven well-known LSCC genes (TP53, ARID1A, NFE2L2, DDR2, KEAP1, PIK3CA, CDKN2A) [6–8], one tumor-associated gene (USH2A) that has not previously been described in LSCC [9], and two genes (DNAH5 and CCDC168) that have not been linked to LSCC previously. TP53 somatic mutations were identified in 62.2% (23/37) of samples, mainly located in the DNA-binding domain (83%). PIK3CA was mutated in 8.1% (3/37) of cases, and these three patients appeared to have poor prognosis. USH2A containing laminin EGF motifs, a pentaxin domain, and many fibronectin type III motifs that are frequently mutated in LSCC, was found to have six missense mutations in our samples.

Frequent inactivating mutations in multiple chromatin-remodeling genes (including *MLL2*, *ARID1A* and *KDM* family), and mutation in one of these genes occurred in almost half of the carcinomas sequenced. Chromatin associated genes are found to be mutated in tumor samples from both never-smokers and smokers. We identified 36 nonsynonymous point mutations, eight stop-gain mutations and seven insertion or deletion mutations in 35 chromatin associated genes, including mutations within KDM4C, MLL2, SETD2, NSD1, PHF2, ZNF408 and ARID1A (see Supplementary Table S3). Frequent mutations in the ARID1A gene have been reported in gastric cancer and ovarian carcinomas [10, 11], which were also detected in smoking and never smoking patients.

### Novel significantly mutated genes within S-LSCC or NS-LSCC

Substantial differences in the mutational burden, spectrum, and affected genes were found between S-LSCC and NS-LSCC (Figure 1B; Supplementary Tables S4 and S5). 16 genes with mutation frequencies all greater than 18% specific to S-LSCC are functionally enriched for gene transcription regulation (Supplementary Table S4). COL3A1 encodes the pro-alpha1 chains of type III collagen, a fibrillar collagen that is found in extensible connective tissues such as skin, lung and the vascular system. Mutations in COL3A1 are associated with Ehlers-Danlos syndrome types IV, and with aortic and arterial aneurysms. COL3A1 is also found to promote cell proliferation, migration, and monocyte recruitment in renal cell carcinoma [12]. STARD13 regulates cytoskeletal reorganization, cell proliferation and cell motility, and acts as a tumor suppressor in breast cancer and colorectal cancer [13]. BAI3 is a p53-target gene that encodes a brain-specific angiogenesis inhibitor, which was preferentially mutated in the smoking group with six point mutations being identified in four patients (Figure 1B; Supplementary Figure S1). These mutations were verified by amplification of the region spanning the mutation point and sequencing (Supplementary Figure S2).

In NS-LSCC 21 genes are identified to contain unique mutations in at least 14% of the samples, but no significant enrichment in biological pathways was identified (Supplementary Tables S5). Among these, cell surface-associated MUC17 functions in epithelial cells to provide cytoprotection, maintain luminal structure, provide signal transduction, and confer anti-adhesive properties upon cancer cells that lose their apical/basal polarization. F-box and WD repeat domain containing-7 (FBXW7) is implicated in multiple cancer types and recently linked to cancer-initiating cells [14]. Moreover, FBXW7 mediates chemotherapeutic sensitivity and its low expression level predicted poor prognosis in non-small cell lung cancer (NSCLC) [15]. Two genes of the ADAM (a disintegrin and metalloprotease domain) family, ADAM18 and ADAM21 are found to be mutated in some of the never-smoking patients. Few studies have been reported on these two genes. However, members of the ADAM family are involved in cancers; for instance, ADAM-12 as a diagnostic marker for the proliferation, migration and invasion in patients with small cell lung cancer [16]. It is worth studying the function of these genes in LSCC.

### Identification of FGF19 as a prognostic marker and potential driver gene in S-LSCC

**Figure 2:**
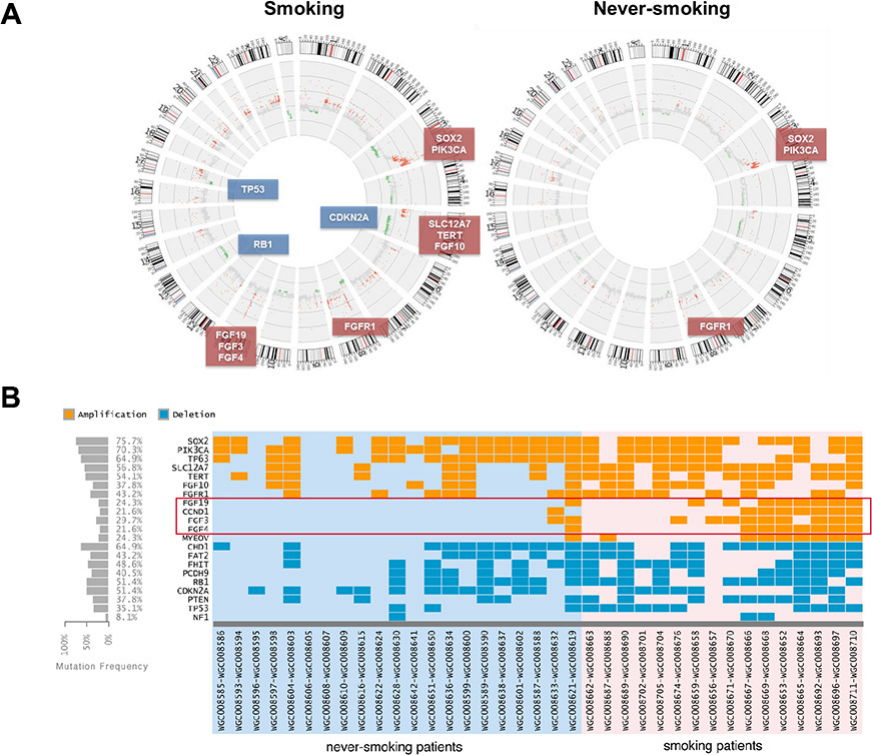
Genomic changes in smokers and never-smokers (**A**) Statistically recurrent peaks of gene amplification and deletion were shown in Circos graphs. (**B**) A heat map of significant different genetic events in 37 LSCC patients is provided for recurrently altered genes between two cohorts in our study. Copy number gains and deletions are color coded according to the legend provided. Each column denotes an individual normal/tumor paired patients, and each row represents a gene.

Somatic copy number alterations (SCNAs) were inferred from sequencing data by read-depth analysis, identifying recurrent peaks of amplification and deletion. Through the comparative analysis between case-matched tumor and adjacent normal tissue exome-seq data, we have identified large-scale chromosome amplification at 3q (SOX2, PIK3CA and TP63), 5p (TERT, SLC12A7 and FGF10), 8q (FGFR1) and 11q (FGF19, FGF3, FGF4 and CCND1), and deletions at 3p (FHIT), 5q (FAT2 and CHD1), 9p21 (CDKN2A), 10q23 (PTEN), 13q (RB1 and PCDH9) and 17p (TP53 and NF1) in both S-LSCC and NS-LSCC (Figure 2A). The previously reported characteristic copy number variations of LSCC, such as the amplifications of SOX2/PIK3CA on chromosome 3 and FGFR1 amplification on chromosome 8 are observed in both patient cohorts (Figure 2A and 2B). For instance, we detected PIK3CA amplifications in 70.2% (26/37) of cases. It is noted that the SOX2 amplification rate of 72.9% in our cohorts is significantly higher than TCGA report (∼23%) [6], but it is accordant to the Korean study (79%) [7].

Interestingly, compared with TCGA and Korean projects [6, 7], our SCNAs landscapes were similar to that in LSCC (gain of 3q, 8q and 11q; loss of 9q21, 10q23 and 17q), but different focal amplification was FGF19, FGF3, FGF4 and CCND1 in 11q [6–8]. The smoker cohort, however, exhibits a much higher level of chromosomal level changes, including both amplifications and deletions, than the non-smoker cohort (Figure 1). In particular, it is interesting to note that a focal amplification region on 11q including multiple FGF ligands (FGF19, FGF3 and FGF4) was preferentially found in smokers (∼40%), in comparison with never-smokers at a frequency of < 5% (1/21) (Figure 2B). CCND1 in the same cluster with FGF19, FGF3 and FGF4, has been found to be amplified in esophageal SCC at 33%, in which the majority are smokers [17]. This rate is comparable to the smoking cohort in our study. It is, however, higher than the Korean study with 16% SCNAs in LSCC [7]. Since we included more NS-LSCC in the cohort, we were able to distinguish such differential SCNAs in smokers vs. never-smokers. RNA sequencing results indicated that FGF19 expression exhibited highest correlation with genomic amplification (R^2^ = 0.663) than other members of FGFs and CCND1 in the 11q cluster. We therefore focused on the characterization of FGF19 in the following studies.

**Figure 3:**
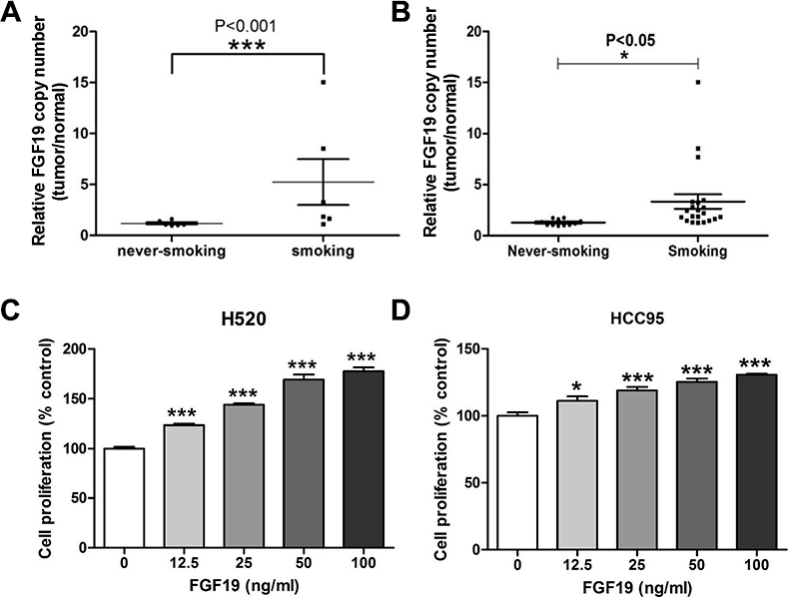
Evaluation of FGF19 as a potential driver gene in LSCC (**A**) Quantitative RT-PCR analysis of FGF19 gene copy number in samples from SQS and SQNS patients. FGF19 expression in tumor sample was normalized to the paired non-tumor samples and data from never-smoking and smoking groups were analyzed (one-way ANOVA, ***p < 0.001). (**B**) Additional validation samples of SQS and SQNS patients were similarly analyzed (one-way ANOVA, *p < 0.05). (**C**–**D**) Cell proliferation assay of LSCC cell lines H520 and HCC95 after FGF19 treatment for 48 h. (one-way ANOVA, *< 0.05, ***p < 0.001).

To further confirm FGF19 amplification, we performed quantitative RT-PCR analysis on selected samples from both cohorts. Indeed, we observed FGF19 gene copy number increase in samples from smoking patients (Figure 3A). The same qRT-PCR analysis was also performed on another set of validation samples that was independently collected (Figure 3B). Validation results indicated that FGF19 was preferentially amplified in smokers than in never-smokers. Excluding the cases with very high amplifications from the comparison study also confirmed FGF19's preferential amplification in the smoker group (Supplementary Figure S3). pFGF19 expression in smokers was also significantly higher than in never-smokers (data not shown). To test the effect of FGF19, we treated two SCC cell lines H520 and HCC95 with increasing concentration of FGF19 for 72 h (Figure 3C and 3D). Apparently exogenous FGF19 significantly promoted LSCC proliferation at a dose-dependent manner. FGF19 stimulated growth of LSCC cells and its amplification associates strongly with smokers suggesting that FGF19 may serve as a druggable driver gene in LSCC, preferentially in smoker patients. These need the further investigation.

FGFs/FGFRs represent an important signaling paradigm in the cell (Supplementary Figure S4). FGF 19 is a hormone-like enterokine released postprandially that has recently emerged as a potential therapeutic agent for metabolic disorders such as diabetes and obesity [18]. Marcelin et al. also demonstrated that FGF19 enhanced the response of AKT phosphorylation to insulin in liver and skeletal muscle [19]. FGF19 has unique specificity for FGFR4, in which the receptor mediates almost all FGF19 activities, with multiple signals at both the N- and C-terminus of FGF19 contributing to FGFR4 activation [20]. Recent data suggest that the FGF19-FGFR4 signaling axis may be a key driver in certain forms of hepatocellular carcinoma (HCC), raising strong interest in therapeutic inhibition of the pathway in this disease setting [21, 22]. FGF19 then acts as an enterohepatic hormone to activate FGFR4 in the liver, which suppresses Cyp7A1 expression resulting in reduced hepatocyte bile acid (BA) synthesis. Binding of hepatic BA to the nuclear hormone receptor FXR in hepatocytes also leads to reduced BA production via induced expression of the atypical nuclear receptor small heterodimeric partner, which cooperates with FGFR4 to suppress Cyp7A1 expression [23]. CYP7A1 is highly up-regulated and has been linked with mechanistic pathways of tobacco-related diseases [24]. The specificity of these amplifications in smoking patients suggests some interesting mechanism of tumor driving mutations in a subset of squamous cell carcinoma.

## CONCLUSIONS

To sum up, this study has comparatively characterized the somatic mutations in S-LSCC and NS-LSCC of Chinese patients, which is an important addition to recent large scale analysis on LSCC. In particular, the higher representation of never smokers in our cohort helps identify key targetable oncogenic mutations in this subtype, such as FGF19. Common and uncommon genetic characteristics in smokers and never-smokers therefore provide invaluable resources for developing novel therapeutic targets in LSCC.

## METHODS

### Tissue acquisition

The collection of human samples and the protocols for the study were approved by the Institutional Ethics Committee of Shanghai Jiao Tong University. All patient-derived samples were collected with informed consents from individuals received surgery as the primary treatment at the Shanghai Chest Hospital between April 2009 and December 2012. Tumor samples were collected immediately following surgical resection, and then kept in pre-cold RPMI-1640 medium with 5% FBS and 1 × Penicillin/Streptomycin, or in Histidine-Tryptophan-Ketoglutarate tissue preservation solution if the estimated shipping time was longer than one hour. All samples were de-identified by the National Tumor Tissue Bank of China before further experiments. Samples were anonymized, and sectioned for further analysis.

### Exome capture, library construction and sequencing

Two independent pathologists reviewed each tumor sample for diagnosis. Genomic DNA was extracted from both tumor and normal tissues using DNeasy Blood and Tissue Kit (Qiagen, CA) according to the manufacturer's protocol. Adaptor-ligated libraries were constructed using TruSeq DNA Sample Prep Kit (Illumina, San Diego, CA). Exome capture libraries were performed with the use of a NimbleGenEZ 44M human Exome Enrichment Kit (NimbleGen). Paired-end 100-bp-long reads were generated using the Illumina HiSeq2000 platform following the standard Illumina protocols. Image analysis, base calling and sequence reads quality assessment were performed using the Illumina Hiseq Control Software (HCS) v1.4.8 with default parameters.

### Sequence mapping and somatic variant detection

All sequence reads were aligned to the NCBI human reference genome (hg19) using the Burrows-Wheeler Aligner (BWA version 0.6.2) software with default parameters. Potential PCR duplicates with the same start site for both ends were removed using Picard (http://picard.sourceforge.net/). To identify potential single-nucleotide variations (SNVs), we performed local realignments using the Genome Analysis Toolkit 2.4 (GATK). VQSR (Variant Quality Score Recalibration) method which builds an adaptive error model using known variant sites was applied to estimate the probability that each variant (SNVs and indels) is a true genetic variant or a machine artifact. Only variants occurring in exons or in canonical splice sites were selected for further somatic variant calling analysis. Variations present in the tumor sample but absent in matched normal tissue were predicted to be somatic. Predicted somatic variations (SNVs and indels) were additionally filtered to include only positions with a minimum of 10 × coverage in both the tumor and matched normal tissue, an observed variant allele frequency of < 3 in the matched normal tissue and that frequency more than 3 in tumor tissue, as well as genotype score as a Phred-scaled confidence at the true genotype > = 40.0. All variants were annotated using the ANNOVAR (functional annotation of genetic variants from high-throughput sequencing data). All somatic mutations were submitted to SIFT version 4.0.5 for predicting whether an amino acid substitution affects protein function. We compared our variants against common and germline polymorphisms present in the dbSNP 137, 1000 Genomes Project (2012 Feb release) and GWAS (June, 2011 NCBI) to discard known germline SNPs. Any sequence variants found in COSMIC v65 were included. For somatic Indel detection, tumor and matched control samples were analyzed with UnifiedGenotyper from GATK and parameter of minIndelFrac was set to 0.05 for getting high sensitivity and avoiding exome probe capture efficiency bias. Events in the tumor were only considered if they were supported by at least four reads. All somatic indel calls were manually reviewed using the Integrative Genomics Viewer (IGV). Indels were annotated as described for SNVs.

### Detection of copy-number alterations

Copy-number alterations were detected using an in house–developed method called exon-specific copy-number (exon-CNV) estimation from sequencing reads depth. Briefly, exon-CNV follows the following procedures: (i) all exon capture regions were merged together if they have overlap. Then, the high GC content's regions like CpG Islands were excluded for the reason of PCR amplification, capture and sequencing bias; (ii) collection of read depth from tumor and normal samples in merged capture regions and calculating the ratios of read depth tumor VS normal; (iii) constructing the probability density function for these ratios, we can identified the highest peak for an example if highest peak was 1.05 so the tumor overall ploidy was 1.05*2; (iv) normalization according to the contamination of the tumor sample with normal cells and according to the tumor overall ploidy; (v) calculation of all copy numbers for the gene segments were analyzed using Control-FREEC, and copy-number alterations were visualized using Circos tools.

### Verification of gene amplification and mutation

To verify driver gene amplification, a quantitative real-time PCR (qRT-PCR) method was designed. In brief, genomic DNA was extracted from tumor or normal tissues by DNA Extraction Kit (TaKaRa) and the DNA quality and concentration was determined with a NanoDrop (Thermo Scientific). Then qRT-PCR was performed on ABI 7900HT by using SYBR Premix Ex TaqII (TaKaRa) with the following primers that span the intron-exon regions of FGF19 (5′-GTGGATTGCTCAGAGCTGCCTG-3′ and 5′-CGGTGCTTCTC GGATCGGTAC-3′). Primers for housekeeping gene RNase P were: 5′-CTGAGTGCG TCCTGTCACTCCAC-3′ and 5′-GAACTCACCTCCCCG AAGCTCAGG-3′. Data were analyzed by using the comparative threshold cycle (Ct) method, and results were expressed as fold differences normalized to RNase P.

To verify gene mutations, primers were designed according to the mutation point. PCR was performed on S1000 Thermal Cycler (BioRad) by using KOD-Plus-Neo (TOYOBO) and the following primers for BAI3 (5′-GGACTGGGTGCTTTACAAGTTTA-3′ and 5′-TAGACTCATCTTATGTTCCCACC-3′). The PCR product was sequenced by GENEWIZ (Suzhou, China), and mutations was identified by BLAST.

### Cell proliferation assays

To examine the effect of exogenous FGF19 on the squamous lung cancer cells, H520 or HCC95 cells (at 3 × 10^3^ cells/well) were plated in 96-well plates. After serum deprivation for 24 h, cells were incubated with increasing concentrations of recombinant FGF19 (R & D Systems) for 72 h in serum-free medium. Viable cell number was determined using the CellTiter 96 Aqueous One Solution Cell Proliferation Assay (Promega) as described by the manufacturer.

## SUPPLEMENTARY MATERIALS FIGURES AND TABLES




