# Supplementary material for: Identification of FGF19 as a prognostic marker and potential driver gene of lung squamous cell carcinomas in Chinese smoking patients
Source: Oncotarget. 2016 Mar 1;7(14):18394–402. doi: 10.18632/oncotarget.7817 (PMC4951296; doi:10.18632/oncotarget.7817)
Supplement: Supplementary file 1 [file oncotarget-07-18394-s001.pdf]

Identification of FGF19 as a prognostic marker and potential driver gene of lung squamous cell carcinomas in Chinese smoking patients

Supplementary Materials

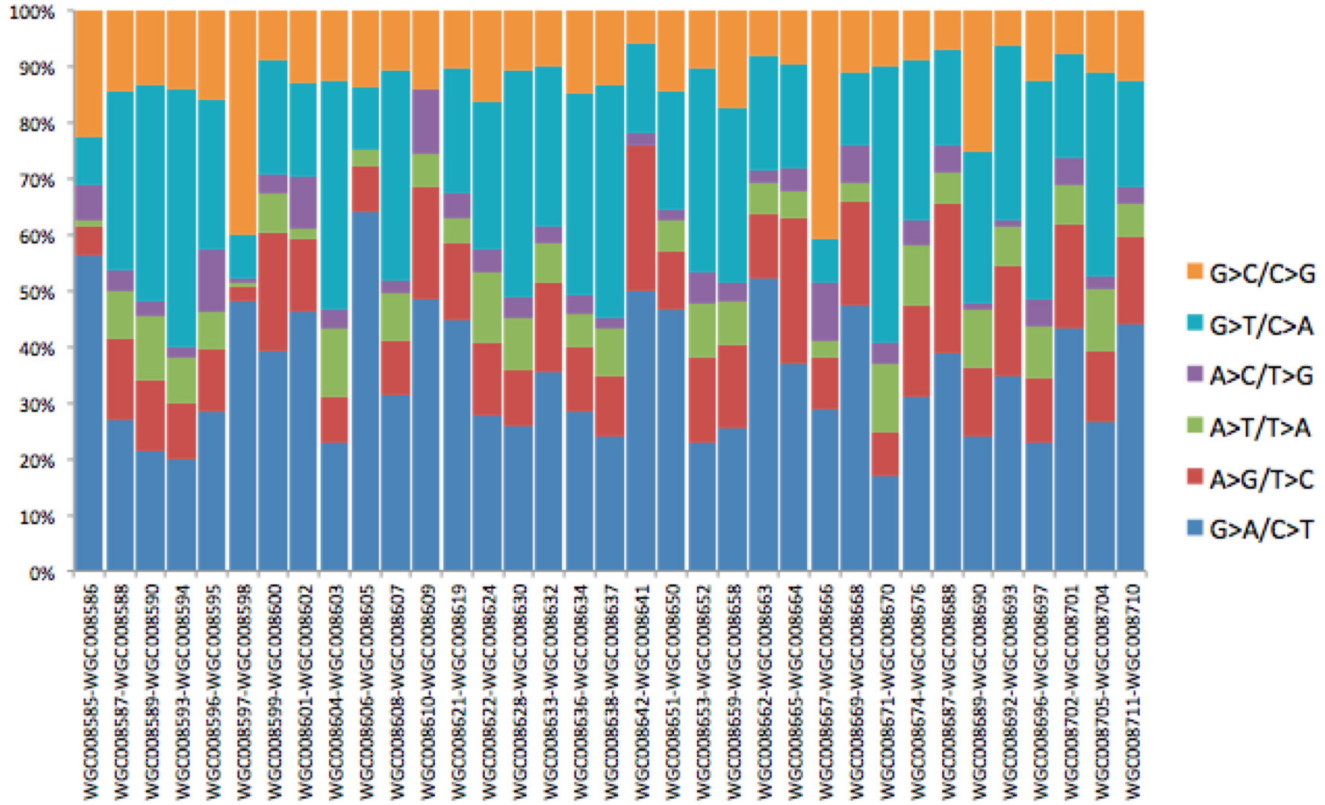

Supplementary Figure S1: The mutation spectrum of six transition (Ti) and transversion (Tv) categories for each patient with point mutations frequency > 20 were shown.

#CHROM	Start	End	REF	ALT	QUAL	Gene location	Gene
chr6	70092744	70092744	A	T	854.19	exonic	BAI3

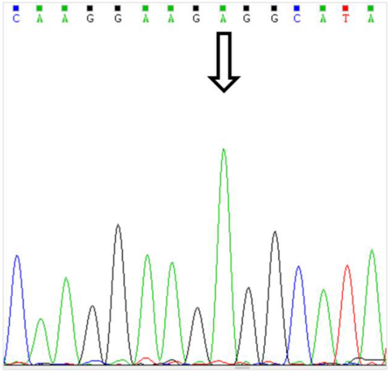

WGC008665: **Normal allele_freq 0.00%**

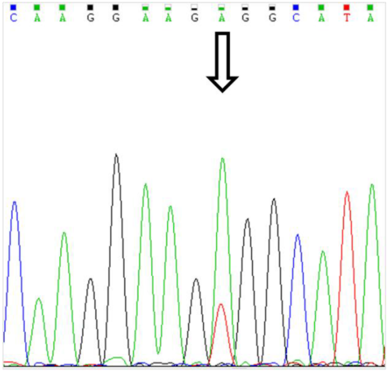

WGC008664: **Tumor allele_freq 35.96%**

#CHROM	Start	End	REF	ALT	QUAL	Gene location	Gene
chr6	69646524	69646524	G	A	1850.19	exonic	BAI3

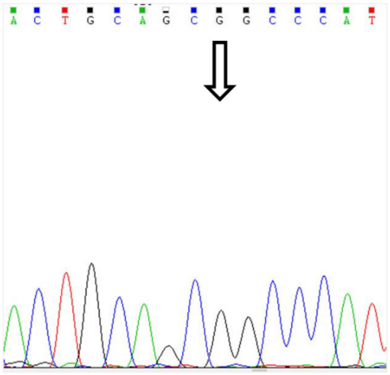

WGC008659: **Normal allele_freq 0.00%**

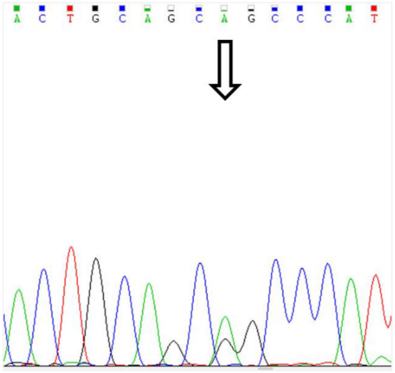

WGC008658: **Tumor allele_freq 56.00%**

#CHROM	Start	End	REF	ALT	QUAL	Gene location	Gene
chr18	14852064	14852064	G	A	1739.19	exonic	ANKRD30B

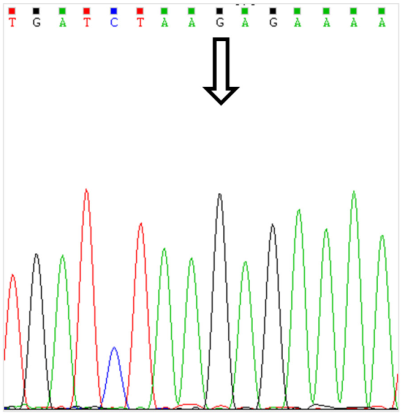

WGC008659: **Normal allele_freq 0.00%**

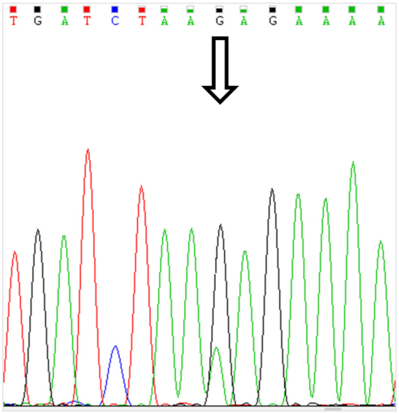

WGC008658: **Tumor allele_freq 38.66%**

Supplementary Figure S2: Verification of point mutations on designated genes in selected samples. Region spanning the SNP on various genes were amplified by PCR and subjected to sequencing. The tumor allele frequency was determined by comparing the area of altered nucleotide vs. the original one in the chromatogram.

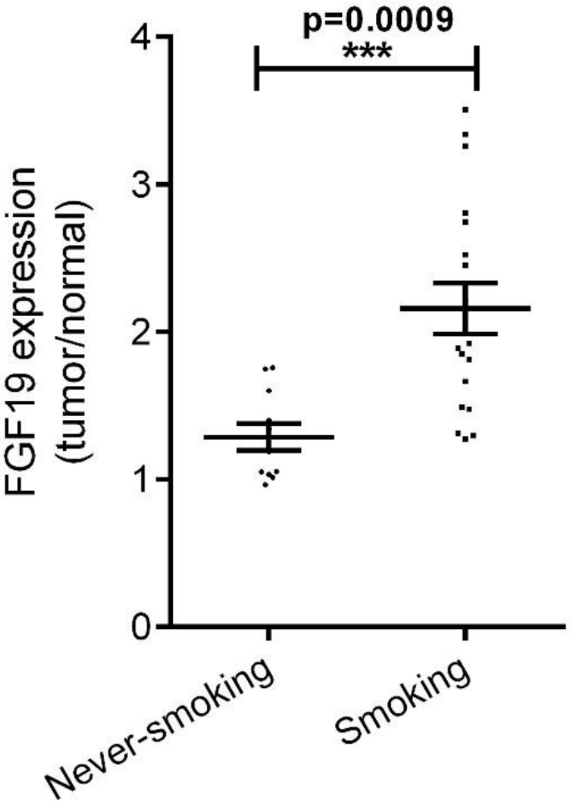

Supplementary Figure S3: Additional validation without those samples with very high FGF19 amplifications of SQS and SQNS patients was analyzed (one-way ANOVA, ******p* = 0.0009).

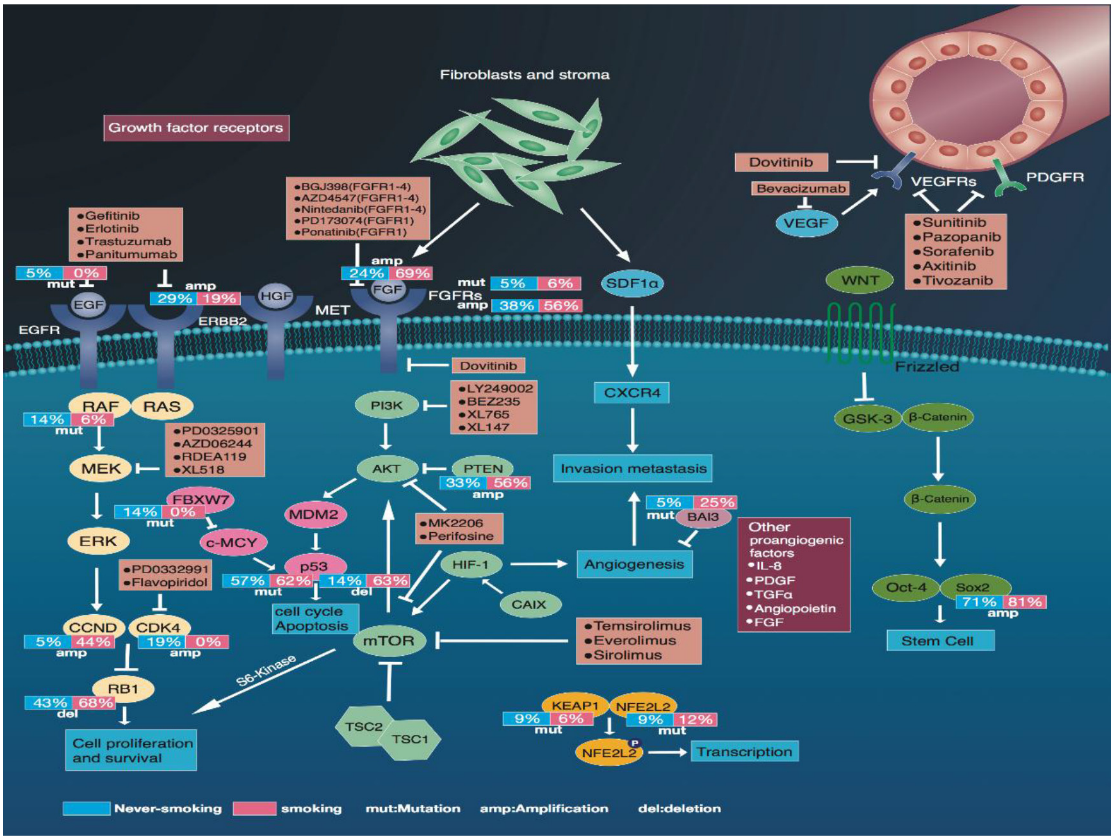

Supplementary Figure S4: Somatically altered pathways in LSCC of smoking and never-smoking patients. Genes are shown along with the percentage of samples with alterations, including gene amplification, mutation and deletion.

Supplementary Table S1: Patient information on smoking status and history, as well as other risk factors

Patient ID	General information			Smoking Informaiton		Age when starting to smoke	Time of exposure to tobacco	Number of cigarettes/ day	Strength to inhale the cigarette	Type of smoke
	Gender	Age	Other risks factors	Family history of lung cancer	Smoking status					
WGC008585	female	58	N/A	no	no	N/A	0	0	0	N/A
WGC008587	male	61	N/A	no	no	N/A	0	0	0	N/A
WGC008589	male	63	N/A	no	no	N/A	0	0	0	N/A
WGC008593	male	56	N/A	no	no	N/A	0	0	0	N/A
WGC008595	female	51	N/A	no	no	N/A	0	0	0	N/A
WGC008597	female	52	right bronchiectasis	no	no	N/A	0	0	0	N/A
WGC008599	male	60	N/A	no	no	N/A	0	0	0	N/A
WGC008601	female	54	N/A	no	no	N/A	0	0	0	N/A
WGC008603	male	74	N/A	no	no	N/A	0	0	0	N/A
WGC008605	male	61	N/A	no	no	N/A	0	0	0	N/A
WGC008607	male	72	N/A	no	no	N/A	0	0	0	N/A
WGC008609	male	71	N/A	no	no	N/A	0	0	0	N/A
WGC008615	female	40	Mitral valve replacement surgery in 2000	no	no	N/A	0	0	0	N/A
WGC008619	male	58	N/A	no	no	N/A	0	0	0	N/A
WGC008622	male	53	N/A	no	no	N/A	0	0	0	N/A
WGC008628	male	58	N/A	no	no	N/A	0	0	0	N/A
WGC008632	male	66	N/A	no	no	N/A	0	0	0	N/A
WGC008634	male	60	N/A	no	no	N/A	0	0	0	N/A
WGC008637	male	66	N/A	no	no	N/A	0	0	0	N/A
WGC008641	male	76	N/A	no	no	N/A	0	0	0	N/A
WGC008650	male	65	N/A	no	no	N/A	0	0	0	N/A
WGC008652	male	54	N/A	no	yes	24	30	40	1200	straw cigarette
WGC008658	male	62	N/A	no	yes	22	40	60	2400	straw cigarette
WGC008662	male	67	N/A	no	yes	18	50	20	1000	straw cigarette
WGC008664	male	63	N/A	no	yes	23	40	40	1600	straw cigarette
WGC008666	male	66	high blood pressure for 20 years	no	yes	17	50	40	2000	straw cigarette
WGC008668	male	56	in 2006, diagnosed with meningeoma	no	yes	25	30	60	1800	straw cigarette
WGC008670	male	65	diabetes mellitus type II	no	yes	30	35	40	1400	straw cigarette
WGC008674	male	68	N/A	no	yes	28	40	25	1000	straw cigarette

WGC008687	male	59	cholecystectomy in 2006	no	yes	29	30	40	1200	straw cigarette
WGC008689	male	60	high blood pressure	no	yes	20	40	40	1600	straw cigarette
WGC008692	male	74	N/A	no	yes	20	60	20	1200	straw cigarette
WGC008696	male	73	bronchiectasis	no	yes	23	50	20	1000	straw cigarette
WGC008701	male	69	N/A	no	yes	25	30	40	1200	straw cigarette
WGC008704	male	70	N/A	no	yes	25	40	30	1200	straw cigarette
WGC008710	male	60	N/A	no	yes	27	30	40	1200	straw cigarette

Supplementary Table S2: Comparing mutation genes identified in our cohort with published data

Genetic abnormality	Gene location	TCGA(Ref.7)	Korean(Ref.9)	Our study
TP53 mut.	17p13.1	80.33%	73%	62.2%
PI3K3CA amp.	3q26.3	32.58%	77%	70.3%
SOX2 amp.	3q26.3-q27	34.26%	79%	75.7%
FGFR1 amp.	8p12	15.17%	31%	43.2%
PTEN mut.	10q23.3	7.86%	11%	5.41%
MET amp.	7q31.1			8.11%
PTEN loss	10q23.3	6.18%	31%	37.8%
KRAS mut.	12p12.1	1.12%		0.00%
LKB1 mut.	19p13.3			2.70%
DDR2 mut.	1q23.3			5.41%
HER2 overexp.	17q11.2-q12;17q21		14%	27.03%
PI3K3CA mut.	3q26.3	16%	9%	8.11
BRAF mut.	7p34	4.49%		2.70%
EGFR mut.	7p12	3.93%	2%	2.70%
AKT1 mut.	14q32.32	1.12%		0.00%
MET mut.	7q31.1			2.70%
HER2 mut.	17q11.2-q12;17q21	3.37%		0.00%

Amplification, amp.; mutation, mut.; overexpression, overexp.

Supplementary Table S3: Mutation genes identified in both smokers and never-smokers

Mutation type	Never-smoking%	Smoking%	Pathway
TP53	57.14%	62.50%	Cell Cycle/Apoptosis
TTN	57.14%	56.25%	other
CSMD3	23.81%	43.75%	other
DNAH5	14.29%	25.00%	other
CCDC168	9.52%	25.00%	other
USH2A	9.52%	25.00%	differentiation
SYNE1	14.29%	25.00%	Cell Cycle
LRP1B	23.81%	18.75%	transport
ZFHX4	19.05%	18.75%	Transcription regulation
OBSCN	23.81%	18.75%	RhoA Pathway
PKHD1L1	19.05%	18.75%	immune response
ARID1A	9.52%	12.50%	Chromatin Modification
NFE2L2	9.52%	12.50%	Cell Cycle/Apoptosis
DDR2	4.76%	6.25%	RTK signaling
KEAP1	9.52%	6.25%	Proteolysis
PIK3CA(mutation)	9.52%	6.25%	PI3K signaling
CDKN2A	4.76%	6.25%	Cell Cycle/Apoptosis
MLL2	9.52%	6.25%	Methyltransferase
SETD2	4.76%	6.25%	Methyltransferase

Supplementary Table S4: Mutation genes identified only in smokers

Gene	Never-smoking%	Smoking%	Pathway
COL3A1	0.00%	25.00%	RhoA Pathway
BAI3	0.00%	25.00%	angiogenesis
ZNF729	0.00%	18.75%	Transcription regulation
ZNF479	0.00%	18.75%	Transcription regulation
ZNF251	0.00%	18.75%	Transcription regulation
TBX22	0.00%	18.75%	Transcription regulation
STARD13	0.00%	18.75%	RhoA Pathway
SSFA2	0.00%	18.75%	other
SPHKAP	0.00%	18.75%	cAMP signal pathway
RIMS2	0.00%	18.75%	cAMP signal pathway
FHL5	0.00%	18.75%	Transcription regulation
DNAJC13	0.00%	18.75%	other
DCC	0.00%	18.75%	Apoptosis
CTNNA2	0.00%	18.75%	adhesion
C1orf168	0.00%	18.75%	other
ANKRD30B	0.00%	18.75%	other

Supplementary Table S5: Mutation genes identified only in never-smokers

Gene	Never-smoking%	Smoking%	pathway
MUC17	23.81%	0.00%	O-linked glycosylation of mucins
RXFP2	19.05%	0.00%	cAMP signal pathway
DOCK10	19.05%	0.00%	RhoA Pathway
AHNAK2	19.05%	0.00%	other
UNC13A	14.29%	0.00%	synaptic vesicle maturation
TNRC18	14.29%	0.00%	Transcription regulation
SPATA31D1	14.29%	0.00%	differentiation
SLITRK5	14.29%	0.00%	synaptic transmission
SCN10A	14.29%	0.00%	cell communication
PRB2	14.29%	0.00%	other
LPHN3	14.29%	0.00%	neuropeptide signaling pathway
IFI44L	14.29%	0.00%	immune response
GRK7	14.29%	0.00%	Chemokine Signaling
FBXW7	14.29%	0.00%	negative regulation of DNA endoreduplication
DAGLB	14.29%	0.00%	neuroblast proliferation
CUX2	14.29%	0.00%	Transcription regulation
CACNA1F	14.29%	0.00%	PKC-Theta Pathway
C5orf42	14.29%	0.00%	other
ADAM21	14.29%	0.00%	spermatogenesis
ADAM18	14.29%	0.00%	spermatogenesis
ABCG2	14.29%	0.00%	ATP catabolic process
